# Can MRI Be Used as a Sole Diagnostic Modality in Determining Clinical Stage in Cervical Cancer?

**DOI:** 10.1093/oncolo/oyac210

**Published:** 2022-10-17

**Authors:** Anke Smits, Maud Steins, Sebastiaan van Koeverden, Stuart Rundle, Heleen Dekker, Petra Zusterzeel

**Affiliations:** Department of Gynecological Oncology, Radboud University Medical Center, Nijmegen, The Netherlands; Department of Gynecological Oncology, Radboud University Medical Center, Nijmegen, The Netherlands; Department of Radiology, Radboud University Medical Center, Nijmegen, The Netherlands; Department of Gynecological Oncology, Queen Elizabeth Hospital, Gateshead, United Kingdom; Department of Radiology, Radboud University Medical Center, Nijmegen, The Netherlands; Department of Gynecological Oncology, Radboud University Medical Center, Nijmegen, The Netherlands

**Keywords:** cervical cancer, MRI, examination under anesthesia, staging, accuracy

## Abstract

**Objective:**

The objective of this study was to compare staging by MRI to clinical staging in patients with cervical cancer and to determine the histological accuracy of staging by MRI and examination under anesthesia (EUA) in early stage disease.

**Methods:**

This was a retrospective cohort study of patients diagnosed with cervical cancer between 2010 and 2020 at the Radboud University Medical Centre, the Netherlands. Pretreatment stage (FIGO 2009) by MRI was compared with staging by EUA. Diagnostic accuracy in terms of sensitivity, specificity, positive, and negative predictive value was calculated for MRI and EUA in patients undergoing surgery (early stage disease) with histological results as a reference standard.

**Results:**

A total of 358 patients were included in the study and MRI-based stage differed from EUA stage in 30.7%. In 12.3% this meant a discrepancy in treatment assignment between MRI and EUA. Diagnostic accuracy of MRI in terms of sensitivity and specificity for detecting early stage disease was comparable to EUA in surgical patients. Further analyses showed that premenopausal status, early stage disease and a tumor diameter of <2 cm were associated with improved comparability of MRI and EUA (98%).

**Conclusion:**

There is still a large discrepancy between MRI and EUA. In patients with suspected early stage disease, diagnostic accuracy of MRI is similar to EUA, especially for premenopausal women with early stage disease and a tumor diameter of <2 cm.

## Teaser

This article compares MRI to examination under anesthesia for the assessment of local disease spread in patients with early stage cervical cancer.

**Implications for Practice**

Diagnostic accuracy of MRI is similar to EUA in patients with suspected early stage disease. Specifically, for premenopausal women with early stage disease and a tumor diameter of <2 cm, MRI may be considered as sole preoperative diagnostic modality to guide treatment.

## Introduction

Due to the relative preponderance of cervical cancer for low-middle income countries and the need to define optimal treatment strategies without recourse to complex imaging modalities, the International Federation of Gynecology and Obstetrics (FIGO) guidelines for cervical cancer staging relies predominantly on clinical examination to ascertain the extent of local disease spread.^1-4^ Tumors that are confined to the cervix (FIGO 2018 stage IA1 to IB2) are usually treated with primary surgery, while treatment for locally advanced stage disease with evidence of spread to the lower vagina or para-cervical tissues (parametrium), as well as the bladder or rectum usually consists of concomitant chemo-radiotherapy.^5,6^ Accurate pretreatment staging is paramount for determining the appropriate treatment modality as surgery followed by chemoradiation may cause unnecessary side-effects and increased morbidity and mortality.^7-10^

Thorough clinical examination for local staging is generally performed under anesthesia (EUA) and consists of inspection, rectovaginal palpation and a cystoscopy or proctoscopy in case of suspicious involvement to adjacent organs. Cross-sectional imaging modalities such as computed tomography (CT) with or without positron emission tomography (PET) are now routinely used in high resource settings to assess loco-regional or distant spread prior to radical treatment.^3,5^ Magnetic resonance imaging (MRI) has recently gained traction as a useful adjunct to clinical examination where available, and is thought to offer greater sensitivity for the assessment of tumor size and parametrial spread compared with clinical examination.^11-16^

Emerging evidence has led to the incorporation of pelvic MRI assessment into the standard staging workup for cervical cancer in The Netherlands.^5^ Following these developments, and with particular regard to the improvements made to MRI imaging and protocols in recent years, the question arises whether both EUA and MRI should remain a standard part of the staging workup of cervical cancers in high resource settings. There are potential disadvantages of continuing this approach in terms of resource allocation and patient risk. Unfortunately, the use of MRI as sole investigative modality for the assessment of local disease in cervical cancer remains to be evaluated.

In this study, we compared the assessment of local disease spread (stage) by MRI to EUA in patients with cervical cancer. In patients with apparent early stage disease undergoing primary surgery, we assessed the accuracy of EUA and MRI compared with final histological outcomes.

## Methods

### Study Population

This was a single-center retrospective cohort study. All patients diagnosed with cervical cancer between 2010 and 2020 in the Radboud University Medical Centre (RUMC), a tertiary level gynecologic oncology center in the Netherlands, were evaluated. All patients had histologically confirmed cervical cancer through biopsy, large loop excision, or conization. Patients were excluded if clinical examination was performed without any anesthesia, MRI was not available, or if the MRI was performed in a non-gynecological oncology center. According to the Central Committee on Research Involving Human Subjects (CCMO), further ethical approval was not required for this study following the Human Research Act.^17^

### Data Collection

Demographic and clinical characteristics were collected retrospectively from medical records. These included age at diagnosis, smoking status, menstrual state, comorbidities, previous surgery, Eastern Cooperative Oncology Group (ECOG) performance status, histological type and grade, FIGO stage, treatment modality and pathological results. Patients staged from 2018 and onward were restaged according to the 2009 classification, enabling comparability. Early stage disease was defined as FIGO 2009 stage IA1, IA2, IB1, and IIA1. EUA and MRI outcomes included clinical and radiological measurement of primary tumor size, extension into surrounding tissues (uterus, parametrium, and vagina), and adjacent organs (bladder and rectum) for all patients.

### Technique

EUA was performed in operating theatres under anesthesia or sedation by 2 gynecologic oncologists and a radiotherapist. MRI was performed with a 3T-scanner at the RUMC using a body- phased array coil. A scan was obtained according to a dedicated protocol for the cervix. The protocol changed during the years due to new insights and technical development. The minimum scan sequences that all patients underwent was a T2-weighted turbo spin-echo (TSE) scan in an axial oblique direction angulated tot the cervix, and a T2 TSE in sagittal and coronal direction. Vaginal and rectal gel was used. In the most recent protocol we obtain also diffusion weighted images witch B-values 50, 400, and 800 in an axial oblique direction of the cervix. Sagittal T1 vibe thin slices 0.9 mm during contrast injection to obtain images with dynamic contrast enhancement followed by a T1 starvibe with a large field of view of the whole pelvis. Final pretreatment stage was determined by the multidisciplinary gynecological oncology team based on findings of both EUA and MRI, after which treatment modality was assigned.

### Outcomes

The primary outcome was the diagnostic value of MRI compared with EUA in terms of pretreatment stage and specifically tumor size, parametrial involvement, vaginal involvement, and adjacent organ involvement. Tumor size was reported in centimeters, but occasionally classified as ≤4 cm versus >4 cm. Treatment allocation, ie, surgery for early stage disease versus chemo-radiotherapy for advanced stage disease, according to MRI and EUA was also compared. Secondary outcomes included factors associated with discordant staging. A subgroup analysis of patients receiving primary surgical treatment was performed (FIGO 2009 stage IA1, IA2, IB1, IIA1), using pathological findings as the reference standard for definitive stage. These included histologically determined tumor diameter and local tumor extensions (uterus, parametrium, vagina, bladder, rectum). Diagnostic accuracy in terms of sensitivity, specificity, positive predictive value (PPV), and negative predictive value (NPV) was calculated for MRI and EUA in patients undergoing surgery with histological results as a reference standard.

### Statistical Analysis

Continuous data were presented as median with ranges and compared using the Mann–Whitney *U* test. Categorical variables were presented as frequencies and compared using the Pearson’s Chi-square and Fisher’s exact test. The accuracy of MRI and EUA for tumor size, and parametrial involvement as evaluated with the calculation of sensitivity, specificity, PPV and NPV. *P*-values were 2-sided and *P < .*05 indicated a statistical significance. Statistical analyses were performed using Statistical Package for the Social Sciences Version 25.0 for Windows (SPSS Inc.).^18^

## Results

**Figure 1.**
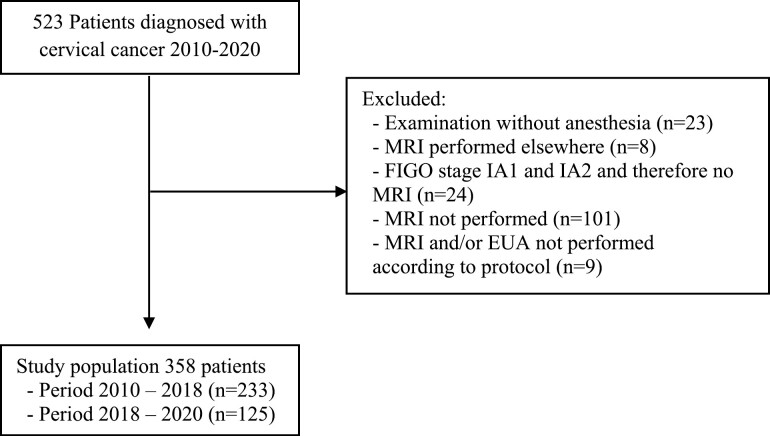
Patient selection process.

A total number of 523 patients were diagnosed with cervical cancer between 2010 and 2020 at the RUMC, The Netherlands. Of these patients, 165 patients were excluded, resulting in a study population of 358 patients (Fig. 1). Early stage disease was defined as stages IA1, IA2, IB1, and IIA1, and advanced stages included stage IB2 and ≥IIA2 (FIGO 2009). Demographic and clinical characteristics of the study population are shown in Table 1. The median age at time of diagnosis was 44 years (range 18–83). Fifty-three percent of patients (*n* = 188) were diagnosed with early stage disease, with the majority being diagnosed with squamous carcinoma (72.6%) or adenocarcinoma (22.1%).

**Table 1.** Demographic and clinical characteristics of study population.

Characteristic	*n* (%)
Age at diagnosis, years, median (range)	44 (18-83)
BMI, median (range)	24.4 (15-51)
Menstrual state	
Premenopausal	241 (67.3%)
Postmenopausal	117 (32.7%)
ECOG score	
0	324 (90.5%)
1	30 (8.4%)
2-4	4 (1.1%)
Comorbidities	
Cardiovascular disease	51 (14.2%)
Chronic pulmonary disease	19 (5.3%)
Inflammatory bowel disease	7 (2.0%)
None	281 (78.5%)
Smoking status	
Yes	117 (32.7%)
No	241 (67.3%)
Stage (FIGO 2009)	
IAI	4 (1.1%)
IA2	5 (1.4%)
IB1	175 (48.9%)
IB2	31 (8.7%)
IIA1	4 (1.1%)
IIA2	4 (1.1%)
IIB	101 (28.2%)
IIIA	7 (2.0%)
IIIB	22 (6.1%)
IVA	5 (1.4%)
Type	
Squamous	260 (72.6%)
Adeno	79 (22.1%)
Adeno-squamous	7 (2.0%)
Others	12 (3.4%)
Grade	
G1	25 (7.0%)
G2	123 (34.4%)
G3	79 (22.1%)
Unknown	131 (36.6%)
Treatment	
Surgery	167 (46.6%)
Chemoradiation	166 (46.4%)
Other^a^	25 (7.0%)

^a^Neoadjuvant chemotherapy followed by surgery as part of the EORTC-55994 study.

### Stage

MRI and EUA assigned the same stage in 248/358 patients (69.3%). Of the 110 patients for whom a difference in stage was noted, MRI gave a more advanced stage than EUA in 58/110 patients (52.7%). In 57 patients, the staging discrepancy was directly related to an assessment of local disease (local tumor spread to parametria, vagina or adjacent organs or tumor size) addressed at EUA. The reasons for local tumor up-staging following MRI scan were: suspicion of early parametrial involvement not detected at EUA (28/58), MRI evidence of vaginal involvement (17/58), extension to pelvic wall (4/58), bladder involvement (2/58), rectum involvement (3/58), or tumor size (3/58). In one further patient a bone metastasis was found. EUA assigned a more advanced stage of disease than MRI in 52 patients based on suspicion of early parametrial involvement not detected at EUA (30/52); MRI evidence of vaginal involvement (8/52); extension to pelvic wall (10/52); bladder involvement (1/52); or tumor size (3/52).

### Tumor Size

Tumor diameter was specified in 332 patients, and MRI and EUA reported a similar tumor size of <2 cm in 93/332 patients (28.0%), of 2–4 cm in 36/332 patients (10.8%) and of >4 cm in 108/332 patients (32.5%), resulting in a concordance rate of 71.4%. Table 2 shows concordance and discrepancy rates of MRI and EUA.

**Table 2.** Tumor size according to MRI and EUA.

EUA	MRI		
	Tumor size < 2 cm	Tumor size 2-4 cm	Tumor size ≥4 cm
Tumor size <2 cm	93 (28.0%)	18 (5.4%)	0 (0%)
Tumor size 2-4 cm	28 (8.4%)	36 (10.8%)	15 (4.5%)
Tumor size ≥4 cm	4 (1.2%)	30 (9.0%)	108 (32.5%)

An additional analysis of 346 patients who had a maximum tumor diameter of <4 cm versus ≥4 cm reported on MRI and EUA showed that MRI and EUA assigned the same tumor size in 297/346 patients (85.8%). EUA showed a tumor of greater than 4 cm in dimension while MRI described a diameter of <4 cm in 20 patients, while in 29 patients MRI suggested a tumor of 4 cm or more, with a combined discrepancy of 14.2%.

### Parametrial Infiltration

In 133/358 patients both EUA and MRI agreed that parametrial infiltration was present. MRI and EUA were discordant in 59 patients with respect to parametrial infiltration. In 28 patients (7.9%) MRI suggested parametrial involvement while this was not reported during EUA. Inversely, EUA reported parametrial involvement in 31 patients (8.8%), which was not seen on MRI.

### Vaginal Involvement

Vaginal involvement was specified in 355 patients by both MRI and EUA, and in 290/355 patients (81.7%) both MRI and EUA agreed on the presence or absence of vaginal infiltration, with vaginal disease present in 75/355 of patients (21.1%). In the 65/355 patients where MRI and EUA differed in terms of vaginal involvement, EUA suggested vaginal disease in 30/355 patients (8.5%) contrary to MRI, while in 45/355 patients (12.7%) MRI suggested infiltrating disease while this was not found through EUA.

### Spread to Adjacent or Distant Organs

Spread to adjacent or distant organs (stage IV disease) was suspected in 11 patients after MRI, and confirmed in 6 patients based on EUA findings. The other 5 patients also had advanced stage disease due to positive nodes (*n* = 4) or parametrial invasion (*n* = 1). All patients with suspected stage IV disease found by EUA were also identified by MRI. The majority of patients (10 of 11) had suspected bladder and/or rectal involvement on MRI, and one patient had suspected bone metastasis.

### Treatment Allocation

The differences between EUA and MRI assessment for local tumor staging would have resulted in allocation to a different treatment group in 44 of 110 patients, which is 12.3% of the total population. Table 3 illustrates reasons for difference in treatment allocation, with surgical management for early stage disease, and chemoradiotherapy for advanced stage disease. If MRI had been the sole staging investigation, 21 patients would classified as advanced stage disease and consequently have received chemo-radiotherapy rather than surgery based on parametrial involvement (*n* = 18), tumor size (*n* = 2), and vagina involvement (*n* = 1). Additionally, if EUA would have been the sole investigation, 23 patients would have been classified as advanced stage disease and received chemo-radiotherapy.

**Table 3.** Concordance and discordance rates of MRI versus EUA in terms of treatment allocation.

	EUA shows early stage disease (IA1, IA2, Ib1, and IIA1)	EUA shows advanced stage disease (IB2 and ≥IIA2)
MRI shows early stage disease (IA1, IA2, Ib1, and IIA1)	169 (47.6%)	Different treatment: 23 (6.4%) Parametrial involvement: 17 Tumor size >4 cm: 4 Vagina involvement: 2
MRI shows advanced stage disease (IB2 and ≥IIA2)	Different treatment: 21 (5.9%) Parametrial involvement: 18 Tumor size >4 cm: 2 Vagina involvement: 1	145 (40.5%)

Further analyses showed that in the group with discordant staging, patients were significantly older (median 50 years (26–82), *P < .*001), and more often postmenopausal (*P = .*001) compared to the group in which MRI and EUA resulted in a concordant stage. In addition, discordant staging was more prevalent with tumor size ≥2 cm (*P* = .03) and locally advanced disease (*P* < .001). Other characteristics such as BMI, performance status (ECOG), tumor type and grade were not associated with staging differences between EUA and MRI (data not shown). In addition, a sub-analysis showed that in a subgroup of premenopausal women with a suspected early stage disease and tumor diameter of <2 cm on MRI (*n* = 108), MRI and EUA were in concordance in 98.1% of patients.

### Histopathological Accuracy

A total of 167 patients underwent primary surgery consisting of radical hysterectomy or radical vaginal trachelectomy with pelvic lymph node dissection (PLND). Of these patients, 163 had suspected early stage disease without lymph node involvement. Three patients with suspected advanced stage disease received primary surgery because of an exophytic growth pattern (FIGO stage 2009 IB2, *n* = 2), inflammatory myofibroblastic tumor type (FIGO stage 2009 IB2, *n* = 1) and another patient received primary surgery due to ambiguity regarding its primary origin; endocervical adenocarcinoma of the cervix versus endometrial carcinoma located low in utero (IIB *n* = 1). In all 4 patients, advanced stage of disease was confirmed by surgico-pathological results. An additional 9 patients were diagnosed with advanced stage disease following surgico-pathological results based on parametrial involvement (*n* = 5) and tumor size (*n* = 4). MRI had suggested this in 2 patients (*n* = 1 tumor >4 cm, *n* = 1 parametrial involvement).

Diagnostic accuracy of MRI and EUA according to histological results is shown in Table 4. Sensitivity and specificity of MRI and EUA for detecting stage of disease were comparable. Six patients had parametrial involvement based on final histology, of which MRI detected 33.3% (*N* = 2). The specificity of MRI for parametrial involvement was 96.3% and was comparable to the specificity of EUA (99.4%). In 7 patients, the tumor had a diameter of more than 4 cm for which MRI and EUA showed comparable sensitivity and specificity rates (Table 4). MRI had a higher sensitivity for measuring tumor size for tumors less than 2 cm compared to EUA.

**Table 4.** Accuracy of EUA and MRI for determining stage, tumor size, and parametrial involvement in patients according pathology.

	Sensitivity	Specificity	PPV	NPV
Early stage disease				
MRI	94.8%	38.5%	94.8%	38.5%
EUA	99.4%	30.8%	94.4%	80.0%
Parametrial involvement				
MRI	33.3%	96.3%	25.0%	97.5%
EUA	16.7%	99.4%	50.0%	97.0%
Tumor size <4 cm				
MRI	99.4%	42.9%	97.5%	75.0%
EUA	100%	42.9%	97.5%	100%
Tumor size <2 cm				
MRI	86.6%	66.7%	89.0%	61.5%
EUA	71.2%	57.9%	84.0%	39.2%
Tumor size 2-4 cm				
MRI	58.1%	84.6%	50.0%	88.4%
EUA	48.4%	73.6%	31.3%	85.1%

Abbreviations: PPV, positive predictive value; NPV, negative predictive value.

## Discussion

The value of MRI in the clinical staging of cervical cancer has received increasing support, with studies suggesting a superior diagnostic accuracy compared with clinical examination such as EUA.^1,2,15,19^ However, its availability in low-resource setting has led to the continuation of a predominantly clinical FIGO staging definition for the determination of treatment allocation. Despite this, the latest FIGO staging classification does allow for the determination of lymph node status to be made on radiological grounds (3). In this study, we compared MRI staging to clinical staging through EUA in primary cervical cancer. In addition, we assessed its accuracy compared to surgico-pathological outcomes to identify patient groups for which MRI may be used as sole diagnostic modality.

Our study showed that in patients with all stage cervical cancer, MRI-based stage differed from EUA in 30.7%, which is similar to that reported in other studies.^19,20^ However, much of this difference related to findings that would not have impacted on treatment allocation. When considering differences between EUA and MRI scan that would have resulted in a change of stage at diagnosis and consequent allocation to a different treatment modality, the difference was much smaller, being 12.3% of patients evaluated. This group includes patients who were evaluated differently by EUA and MRI scan with respect to parametrial involvement (16.7%), vaginal involvement (21.1%) and a difference in tumor size exceeding 4 centimeters, with a slight trend toward a larger tumor delineation by MRI. In addition, we have identified that in premenopausal patients with early stage disease and a tumor of less than 2 cm on MRI, assigned stage and consequent treatment modality concurs in 98% with EUA. MRI may therefore be regarded as an acceptable sole diagnostic mean for assigning surgical treatment to this group of patients, particularly considering the known limitations of clinical examination.^1,2^

In our study, MRI confirmed IV stage disease in all women with suspected stage IV disease based on EUA. Despite the limited number of patients, our findings concur with previous studies stating that MRI reliably excludes the presence of bladder and rectal invasion.^15,21^ In addition, a secondary analysis of the EMBRACE study by Knoth et al. states that MRI overestimates incidence of bladder infiltration but that MRI may better visualize different histological layers of the bladder wall and therefore may even be superior to cystoscopy during clinical examination.^20^ However, as MRI may overestimate, EUA should not be omitted when advanced stage is suspected solely based on bladder and/or rectal involvement.

Determining the value of MRI in cervical cancer is partly impeded by the absence of a histological reference standard in advanced stage disease, as these patients receive chemo-radiotherapy therapy as primary treatment. In addition, international standardized cervix protocols and current guidelines on how to weigh discrepant findings between MRI and clinical examination are lacking. At our institution, we experienced that in case of discrepancy, EUA findings outweighed MRI findings in the majority of cases when assigning treatment modality.

Therefore, delineating the accuracy of MRI, and consecutively identifying patients for which MRI alone is sufficient in determining stage and treatment strategies, can only be done in patients receiving surgical treatment. In this study, we found that the diagnostic accuracy of MRI in terms of sensitivity and specificity for detecting early stage disease was comparable to EUA, with respective rates of 94.8% and 38.5% for MRI. Other studies have reported sensitivities ranging from 66.7% to 93.0% and specificities ranging from 53.0% to 98.0%.^15,19,22^ Thomeer et al systematically reviewed the diagnostic value of MRI and EUA, and concluded that MRI was comparable to EUA in staging early stage disease with pooled sensitivities of respectively 93.0% and 97.0%. In addition, they found a specificity which was higher for MRI than for EUA (79.0% vs 53.0%).^1^ Despite demonstrating the non-inferiority of MRI to EUA in our study, we may attribute the difference in reported specificity to our study size. In addition, differences in sensitivity may be explained by the differences in MRI protocols between studies as not all studies used fat-suppressed scans and diffusion-weighted images (DWI). Previous studies showed that DWI improves staging accuracy by a more precise tumor measurement, detection of parametrial involvement and lymph nodes metastases.^13,14,23,24^ Furthermore, MRI techniques like fast-spin echo (FSE), contrast-enhanced images, the use of a higher magnetic field and the use of a body phased array also improve the diagnostic accuracy of MRI and were part of the cervix protocol used at our institution.^1^

Specificity of MRI and EUA for parametrial involvement in our study were comparably high (96.3% and 99.4%). Correct assessment of the parametria is essential as parametrial involvement alters the treatment modality. Our findings are supported by other studies which reported similar specificity rates when comparing MRI and clinical examination.^1,15^ Interestingly, other studies have stated that MRI even has a higher sensitivity than EUA, and thereby is superior to EUA in identifying parametrial involvement, with pooled sensitivity rates of 84% compared to 40% sensitivity of clinical examination.^1,14,15,25^

We showed that MRI had comparable sensitivity to EUA in predicting tumor size for tumors smaller than 4 cm, correctly identifying patients suitable for surgical treatment based on tumor size. Other studies which also showed higher diagnostic value of MRI in estimating tumor size, especially for tumors with a diameter less than 2 cm.^2,26,27^

Strengths of our study include the clinical staging process which was performed by gynecological oncologists and radiotherapists with vast experience, ensuring homogeneity of our results. In addition, MRIs were assessed using a standardized cervix protocol throughout the whole study period and were assessed by experienced radiologists. Furthermore, we have reported one of the largest series to date in a high resource setting of all patients with stage cervical cancer undergoing surgery or chemo-radiotherapy.

Main limitations of this study include its retrospective and single-institution design. Secondly, the combination of EUA and MRI imaging (according to protocol) was not available for 141 patients which may have resulted in a selection-bias. In addition, as these are the results of a tertiary oncology center in The Netherlands, we believe these results and conclusions may not be extrapolated to low resource settings with inconsistent availability and limited techniques of MRI.

Despite the fact that MRI is shown to be comparable to EUA in detecting early-stage disease, and even superior in detecting tumor size and parametrial involvement, there have been few studies advocating its sole usage in pretreatment staging of cervical cancer. The reluctance of proposing MRI as the standard sole diagnostic modality in determining clinical stage for suspected early stage cervical cancer may be partly explained by its availability in low-resource settings. While recognizing these global disparities, advocating the additional value of advanced imaging may encourage access and incorporation of these techniques into the care of cervical cancer patients worldwide. In high resource settings such as The Netherlands, patients with cervical cancer are referred to tertiary Gynecological Oncology centers where these prerequisites can be met. The recent revision of FIGO 2018 staging system further prompts the use of MRI to evaluate lymph node involvement as it is now an integral part of staging.^3^ Therefore, MRI should be further explored as sole diagnostic modality in young women with suspected early stage disease and a tumor diameter under 2 cm.

However, there may be certain exceptions, such as an indication for radiotherapy based on histology, ineligibility for surgery, or if further assessment of cervical length is required in women eligible for fertility-preserving surgery.^6^ Furthermore, in case of suspected isolated vaginal involvement on MRI or suspected inflammation or edema secondary to previous biopsy impeding tumor delineation by MRI, recourse to clinical examination might still be indicated for confirmation to avoid inappropriate treatment allocation and unnecessarily limiting treatment options.^23^

## Conclusion

As cervical cancer is still the second most prevalent cancer in low- and middle-income countries, clinical examination remains the cornerstone of treatment allocation.^3,4^ There is still a significant discrepancy between MRI and EUA, specifically for suspected advanced stage disease in the absence of a histological reference. In this study, we found that the diagnostic accuracy of MRI in terms of sensitivity and specificity for detecting early stage disease was comparable to EUA in surgical patients. Further analyses showed that premenopausal status, early stage disease and a tumor diameter of less than 2 cm (FIGO 2018 stage 1BI) were associated with improved comparability of MRI and EUA (98%).

Future studies should focus on further identifying appropriate patient groups, and assessing MRI as sole diagnostic strategy by evaluating treatment and survival outcomes. In addition, we recommend the incorporation of a standard format for evaluation of cervical cancer stage on MRI which will aid uniformity and comparability of results.

## Data Availability

All data generated or analyzed during this study are included in this published article.
